# Key role of singlet oxygen and peroxynitrite in viral RNA damage during virucidal effect of plasma torch on feline calicivirus

**DOI:** 10.1038/s41598-018-36779-1

**Published:** 2018-12-18

**Authors:** Risa Yamashiro, Tatsuya Misawa, Akikazu Sakudo

**Affiliations:** 1 Laboratory of Biometabolic Chemistry, School of Health Sciences, University of the Ryukyus, Nishihara, Okinawa, 903-0215 Japan; 2 Department of Electrical and Electronic Engineering, Faculty of Science and Engineering, Saga University, Saga, 840-8502 Japan

## Abstract

A dielectric barrier discharge (DBD) plasma torch has been used to evaluate the mechanism underlying inactivation of feline calicivirus (FCV) by plasma treatment. Plasma treatment of cell lysate infected with FCV F9 strain reduced the viral titer of the median tissue culture infectious dose (TCID_50_). The *D* value (treatment time required to lower the viral titer to 1/10) was 0.450 min, while the viral titer dropped below the detection limit within 2 min. FCV was not significantly inactivated by heat or UV applied at levels corresponding to those generated from the DBD plasma torch after 2 min (38.4 °C and 46.79 mJ/cm^2^ UV, respectively). However, TCID_50_ was reduced by 2.47 log after exposure to 4.62 mM ONOO^−^, corresponding to the concentration generated after 2 min of plasma treatment. Radical scavengers, including superoxide dismutase, dimethyl sulfoxide, and catalase, did not significantly affect viral titers; however, sodium azide, uric acid, and ascorbic acid, which are scavengers of ^1^O_2_ radicals, ONOO^−^, and peroxynitrous acid (ONOOH; produced from ONOO^−^ under acidic conditions), respectively, significantly increased TCID_50_ and intact viral RNA. These findings suggest that ONOO^−^ and ^1^O_2_ play an important role in FCV inactivation by attacking viral RNA during DBD plasma torch treatment.

## Introduction

Norovirus is a foodborne agent for infectious gastroenteritis and a major cause of food poisoning^1^. Norovirus causes 19–21 million cases of acute gastroenteritis (inflammation of the stomach or intestine or both) annually in the United States and 570–800 deaths, mostly among young children and the eldery^2^. Incubation time is estimated to be 12–72 h (mainly 24–48 h)^3^. A very small number (18–100) of virions is sufficient to infect humans^4^ and norovirus can survive for prolonged periods of time under normal environmental conditions^5^. Oysters and other shellfish are an important vehicle for the transmission of norovirus, while secondary infection may occur via contact with contaminated surfaces such as door knobs and table tops. During an outbreak of norovirus, strict regimes for washing/disinfection of foods and food contact surfaces must be implemented prior to preparing and consuming foods as well as the observance of hand washing/disinfection/sanitization^6^.

Chlorine bleach solution with a concentration of 1000–5000 ppm or other disinfectants registered as effective against norovirus by the US Environmental Protection Agency (US-EPA)^7^ are generally recommended for the inactivation of norovirus^8^. However, the use of these chemical disinfectants can cause problems because they may act as irritants, lead to the generation of toxic gas or result in metal corrosion. In addition, the inactivation efficiency of these reagents changes under different conditions including pH, concentration, exposure time and reaction temperature, and especially by the presence of contaminants such as organic matter^5,9–11^.

There are many disinfection technologies including thermal treatments such as autoclaving, steam pasteurization, ohmic heating, and high frequency heating^12^. However, human norovirus is relatively resistant to heat and can survive temperatures as high as 60 °C (140 °F)^13^. Furthermore, these thermal disinfection procedures can lead to nutritional loss and have an adverse effect on the food characteristics.

Recently, there has been research into the development of non-thermal disinfection methods for foods including ozone^14,15^, UV radiation^16^, X- and γ-rays^17^, pulsed light^18^, high pressure^19^, pulsed electric field^20^, oscillating magnetic field^21^ and ultrasonic processing^22^. However, these methods are hindered by both their excessive set-up costs and the need for trained personnel. In summary, there are no methods for the effective non-thermal inactivation of foodborne pathogens, especially human norovirus, that have completely satisfied all criteria, such as being non-toxic, non-irritant and economically viable.

We have recently studied plasma technology as an innovative disinfection methodology^12^. Plasma is commonly referred to as the fourth state of matter after solid, liquid and gas. Plasma has been shown to be effective for the inactivation of bacteria, such as *Salmonella*, and various viruses, such as influenza virus as well as adenovirus, under non-thermal conditions^23–26^. Plasma produces ultraviolet (UV) radiation, an electric field and various reactive oxygen species (ROS) and reactive nitrogen species (RNS), which are thought to mainly contribute to the mechanisms of inactivation. However, whether ROS and RNS are essential for the virucidal effect of plasma remains unclear.

Here, we examined plasma-induced inactivation of feline calicivirus (FCV) as a surrogate system for human norovirus, because an *in vitro* proliferation method for this virus has not been established^27,28^. Recently, *in vitro* models for FCV proliferation using B cells^29^ and enteroids^30,31^ have been reported, which both require sophisticated techniques. Furthermore, the US-EPA^32^ and other studies^33,34^ have previously used FCV as a surrogate of human norovirus. FCV belongs to the same family of *Caliciviridae* as human norovirus and shows similar characteristics^35^. In addition, FCV has the most accumulated data among surrogate viruses. Moreover, FCV displays greater resistance to various chemical and physical treatments than murine norovirus, a similar surrogate of human norovirus^36–38^, suggesting that FCV is an appropriate surrogate of norovirus for inactivation studies.

Recently, we have developed a dielectric barrier discharge (DBD) gas plasma torch composed of a ceramic tube, stainless steel mesh, and copper plate that delivers a high-voltage and high frequency pulse to air using a power supply to generate gas plasma^39^. The DBD plasma torch was tested to establish whether it could be used to disinfect a suspension of FCV. Furthermore, to clarify the disinfection mechanism of FCV by the DBD plasma torch, potential changes to genomic RNA after DBD plasma treatment were investigated. In addition, disinfection factors that may be responsible for the observed virucidal activity of the DBD plasma torch were analysed including the generation of heat, long-wave ultraviolet radiation (UV-A), hydrogen peroxide (H_2_O_2_), and peroxynitrite (ONOO^−^) as well as other ROS and RNS. In order to establish which of these factors is/are particularly important for disinfection of FCV, we subjected the FCV to each of the disinfection factors independently to determine the relative contribution of each factor in turn. We also analyzed the action of the DBD plasma in combination with radical scavengers. Finally, the virucidal action of the plasma treatment is discussed.

## Results

**Figure 1:**
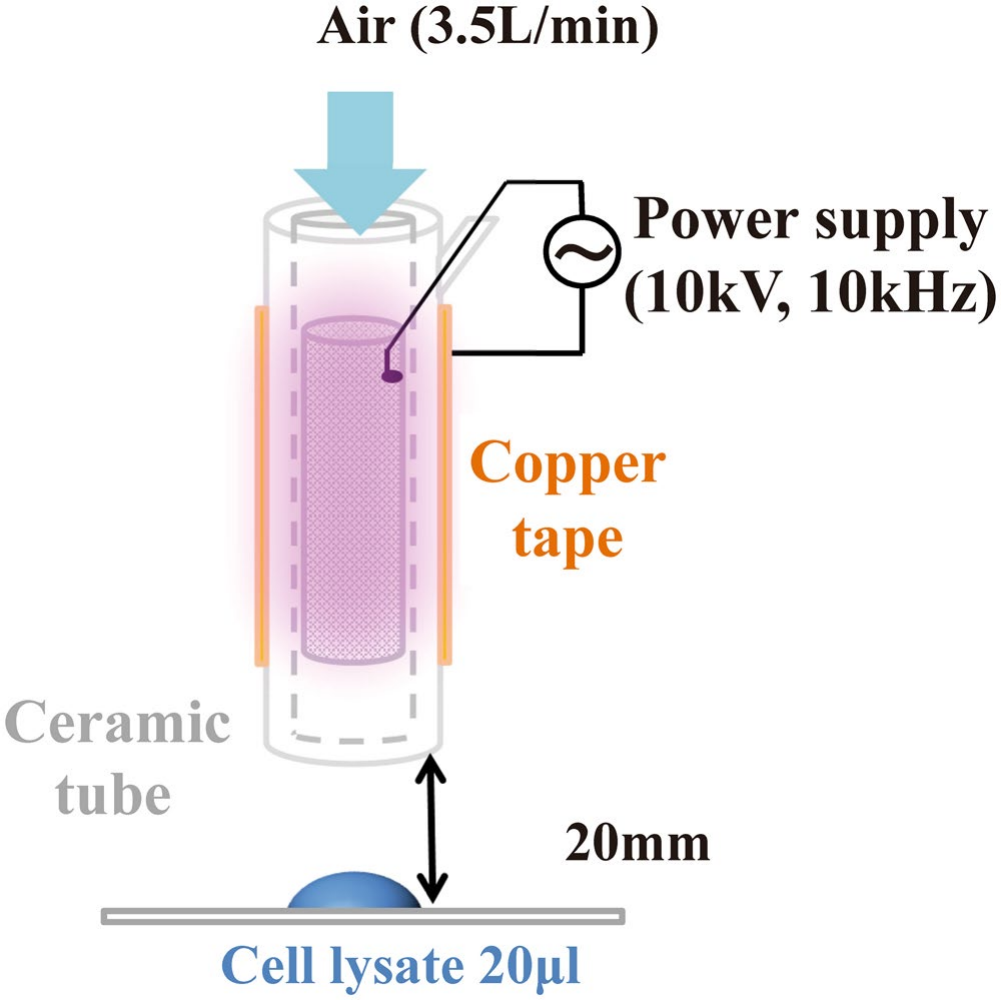
Schematic representation of the dielectric barrier discharge (DBD) plasma torch. The DBD plasma torch comprises a ceramic tube (Al_2_O_3_), containing stainless-steel mesh, covered with copper tape. The copper tape and stainless-steel mesh were connected to a power supply (10 kV, 10 kHz). The air flow rate was maintained at 3.5 L/min using an air pump during gas plasma generation. The distance from the tip of the plasma torch to the liquid surface on the cover glass was 20 mm.

**Figure 2:**
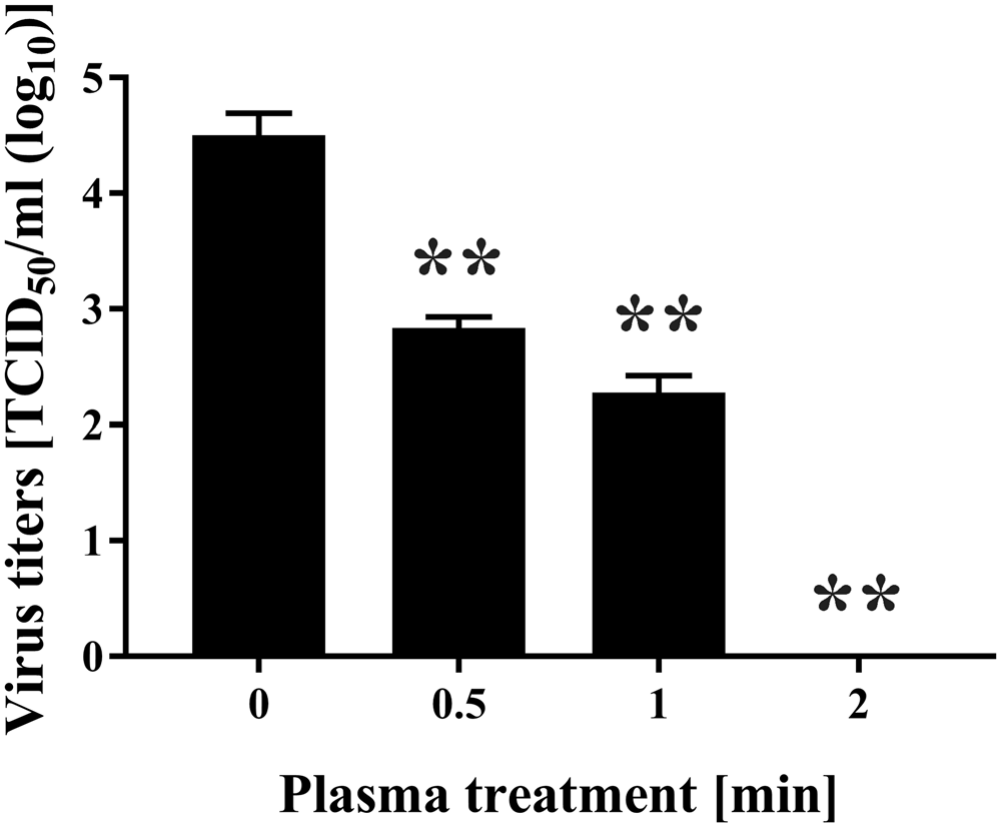
Treatment with the DBD plasma torch reduced viral titer of feline calicivirus (FCV). The FCV-infected cell lysate was exposed to the DBD plasma torch for the indicated time (min). Viral titer of FCV [TCID_50_ (50% tissue culture infective dose)/ml] was calculated after DBD plasma treatment as described in Materials and Methods. Zero in virus titer means below the detectable limit. Differences where **p < 0.01 versus control (0 min) were considered significant.

Firstly, the change of FCV infectivity after treatment with the DBD plasma torch was investigated. A 20 μL aliquot of virus-infected cell lysate was dropped onto a cover glass and then exposed to the DBD plasma torch for 0, 0.5, 1 or 2 min (Fig. 1). After plasma treatment the suspension was recovered with 200 μL of PBS and the TCID_50_ determined (Fig. 2). The initial viral titer of FCV (0 min) was 3.81 × 10^4^ ± 1.58 × 10^3^ TCID_50_/ml. Plasma treatment caused a significant decrease in viral titer: 7.15 × 10^2^ ± 1.56 × 10^2^ TCID_50_/ml at 0.5 min; 2.11 × 10^2^ ± 6.25 × 10^1^ TCID_50_/ml at 1 min; below the detection limit at 2 min. From the data, we calculated the *D* value, which is the time required to achieve 90% reduction of viral titer. The *D* value calculated from the slope within 1-min of DBD plasma treatment was 0.450 min.

**Figure 3:**
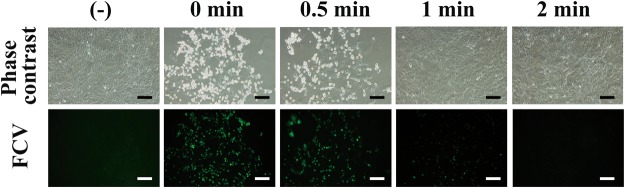
Immunofluorescent assay revealed a decrease of FCV infectivity after DBD plasma treatment. The FCV-infected cell lysate was subjected to DBD plasma treatment for 0, 0.5, 1, and 2 min. Recovered lysate was added to Candell-Rees feline kidney (CRFK) cells and after culturing for 1 day the cells were fixed with glutaraldehyde and analyzed by an immunofluorescent assay using anti-FCV antibody. Uninfected control (−) was included. Phase contrast images (exposure time 1/7 sec) and the results of the immunofluorescent assay for FCV (Green) (exposure time 4 sec) are shown. The scale bars indicate 100 µm.

Next, an immunofluorescent assay using anti-FCV antibody against FCV capsid protein was performed after incubation of CRFK cells with the plasma-treated FCV (Fig. 3). A proliferation of FCV in CRFK cells was observed after incubation with untreated cell lysate (0 min) for 1 day. However, reduced FCV proliferation was seen in CRFK cells after incubation with cell lysate exposed to plasma in a treatment time-dependent manner. These findings suggested that the DBD plasma torch treatment decreased infectivity of FCV.

**Figure 4:**
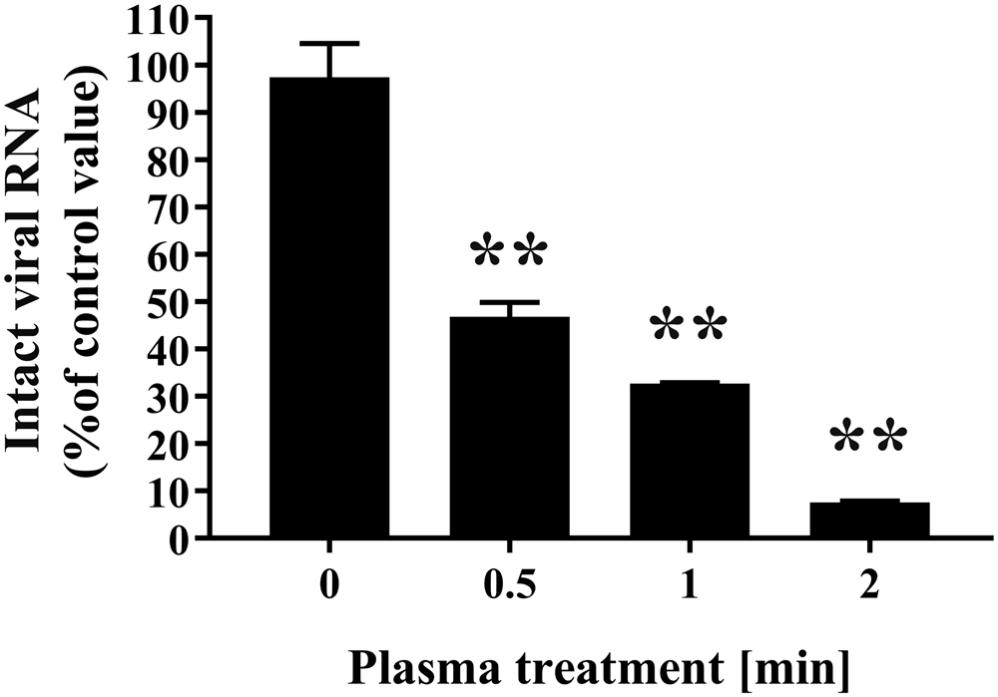
Quantitative analysis of FCV RNA after DBD plasma torch treatment. FCV-infected cell lysate was subjected to DBD plasma torch treatment for the indicated time (min). Extracted viral RNA was then analyzed to compare the levels of intact viral RNA by real-time polymerase chain reaction (PCR) using primers for FCV (FCV-F1 and FCV-R1) as described in Materials and Methods. Differences where **p < 0.01 versus control (0 min) were considered significant.

To further investigate the effect of DBD plasma-treatment on FCV, biochemical changes to the viral RNA were analyzed. Real-time PCR was carried out by using sequence-specific primer sets for FCV including FCV-F1 and FCV-R1 (Fig. 4). The results confirmed the detection of intact FCV RNA by PCR amplification in the untreated FCV sample (0 min). The specificity of the real-time PCR was confirmed by dissociation curve analysis of the reaction products. Significantly reduced levels of intact FCV RNA were found in DBD plasma torch treated FCV for 0.5, 1, and 2 min by comparison with the untreated FCV (0 min).

**Figure 5:**
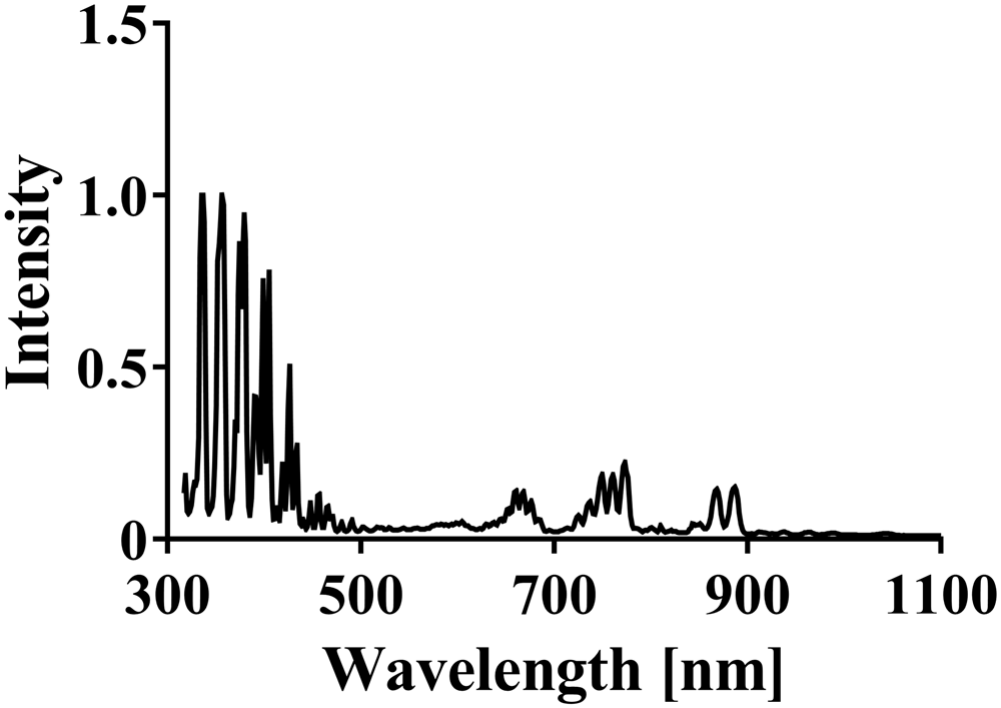
Spectrum of emission during operation of the DBD plasma torch. The optical emission spectrum was analyzed using a MCPD-7000 spectrophotometer (Otsuka Electronics Co. Ltd.) during operation of the DBD plasma torch.

Next, we investigated inactivating factors generated from the DBD plasma treatment. Firstly, UV produced during operation of the plasma torch was analysed using UV label-S for UV (Table 1). The energy of the UV was estimated from the change in colour of the corresponding test strip, which was scanned and compared with a calibration curve of reference standards. The amount of UV radiation to which the samples were exposed increased in a time-dependent manner. The energy values were estimated as follows: 4.59 ± 0.58 mJ/cm^2^ at 0 min; 15.14 ± 0.51 mJ/cm^2^ at 0.5 min, 25.69 ± 0.52 mJ/cm^2^ at 1 min; 46.79 ± 0.76 mJ/cm^2^ at 2 min. Analysis of the emission spectrum (300–1100 nm) of the DBD torch indicated that UV-A (320 to 400 nm) is generated during plasma production (Fig. 5). Further analysis of these spectra showed strong peaks around 300–500 nm, possibly due to N_2_ emission of the second positive system (C^3^Πu – B^3^Πg), and very weak peaks around 650–900 nm, possibly due to the N_2_ first positive system (B^3^Πg – A^3^Σu^+^)^40^. These results are consistent with our previous findings at 200–800 nm using another spectrophotometer (S-241, Soma Optics Ltd., Tokyo, Japan)^39^.

**Table 1:** Generation of UV, heat, ONOO^−^, and H_2_O_2_ during operation of the DBD plasma torch.

Time [min]	UV [mJ/cm^2^]	Temperature [°C]	ONOO^−^ [mM]	H_2_O_2_ [mg/L]
0	4.59 ± 0.58	26.67 ± 0.17	0.18 ± 0.00	11.69 ± 0.31
0.5	15.14 ± 0.51	34.13 ± 0.12	1.69 ± 0.00	67.56 ± 3.67
1	25.69 ± 0.52	36.30 ± 0.20	2.33 ± 0.00	79.32 ± 3.19
2	46.79 ± 0.76	38.40 ± 0.12	4.62 ± 0.00	87.10 ± 3.29

Next, the temperature on the surface of the sample liquid was measured using infrared thermography FLiR i5 (FLIR Systems) (Table 1). The surface temperature of the FCV-infected cell lysate was 26.67 ± 0.17 °C at 0 min, 34.13 ± 0.12 °C at 0.5 min, 36.30 ± 0.20 °C at 1 min, and 38.40 ± 0.12 °C at 2 min.

Next, the production of ONOO^−^ during the operation of DBD plasma torch was measured using the Griess method. The concentration of ONOO^−^ was 0.18 ± 0.00 mM at 0 min, 1.69 ± 0.00 mM at 0.5 min, 2.33 ± 0.00 mM at 1 min, and 4.62 ± 0.00 mM at 2 min. Semi-quantitative measurement of H_2_O_2_ was performed using a chemical indicator kit (Quantofix peroxide 100 test strip). Plasma torch treatment of PBS of the chemical indicator strip was performed at a distance of 20 mm from the torch tip to the indicator for 0, 0.5, 1, 2, and 5 min. The concentration of H_2_O_2_ detected by the strip was 11.69 ± 0.31 mg/L at 0 min, 67.56 ± 3.67 mg/L at 0.5 min, 79.32 ± 3.19 mg/L at 1 min, and 87.10 ± 3.29 mg/L at 2 min.

**Figure 6:**
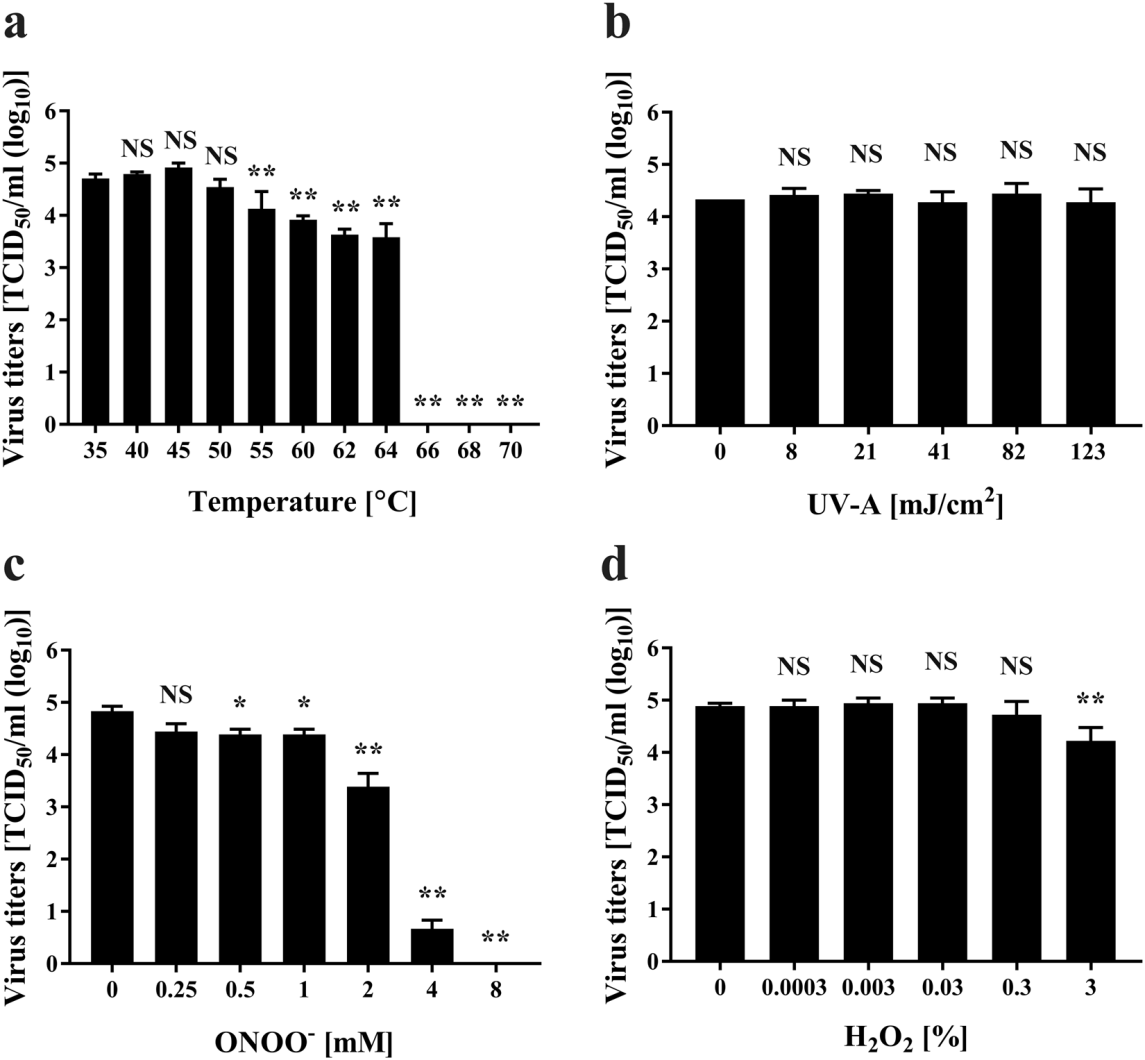
Stability of FCV under various conditions. After heat treatment (35–68 °C) for 2 min (**a**), UV-A treatment at the indicated energy (mJ/cm^2^) for the indicated time (0–30 min) using a UVGL-58 device (UVP) (**b**), treatment with peroxynitrite (ONOO^−^) (0–8 mM) for 2 min (**c**), treatment with hydrogen peroxide (H_2_O_2_) (0–3%) for 2 min (**d**), the FCV-infected cell lysate was subjected to viral titration assay to determine viral titer (TCID_50_/ml). Refer to Materials and Methods for detailed descriptions of the protocols. Zero in virus titer means below the detectable limit. Differences where *p < 0.05 and **p < 0.01 versus control (35 °C for temperature, 0 mJ/cm^2^ for UV-A, 0 mM for ONOO^−^, and 0% for H_2_O_2_) were considered significant, while NS means no significance.

Next, we aimed to analyze which of these virucidal factors was primarily responsible for the observed inactivation of FCV. This was achieved by separately exposing FCV to heat, UV-A, ONOO^−^ or H_2_O_2_. FCV cell lysate treated with heat showed no significant changes in viral titer (TCID_50_/m) after treatment at 40–50 °C for 2 min compared to control (35 °C), whereas a significant reduction of viral titer was observed after treatment at 55–70 °C for 2 min (Fig. 6A). Treatment at 66 °C for 2 min reduced the viral titer to below the detectable limit. The results suggested that FCV is inactivated after heating above 55 °C.

Spectral analysis of emitted radiation during operation of the DBD plasma torch showed it to be mainly UV-A. Thus, FCV cell lysates were treated with UV-A and then subjected to viral titration (Fig. 6B). The energy of UV-A emitted by the plasma torch was estimated using UV label-S. The results showed no significant reduction in viral titer after UV-A exposure of up to 123 mJ/cm^2^.

Treatment of FCV-infected cell lysate with ONOO^−^ for 2 min significantly reduced the virus titer from 7.15 × 10^4^ ± 1.56 × 10^4^ TCID_50_/ml (0 mM ONOO^−^) to 3.09 × 10^4^ ± 9.17 × 10^3^ TCID_50_/ml at 0.25 mM ONOO^−^, 2.49 × 10^4^ ± 3.36 × 10^3^ TCID_50_/ml at 0.5 mM ONOO^−^, 2.49 × 10^4^ ± 3.36 × 10^3^ TCID_50_/ml at 1 mM ONOO^−^, 2.75 × 10^3^ ± 9.64 × 10^2^ TCID_50_/ml at 2 mM ONOO^−^, and 4.87 × 10^0^ ± 1.06 × 10^0^ TCID_50_/ml at 4 mM ONOO^−^. The virus titer was below the detection limit after treatment with 8 mM ONOO^−^ (Fig. 6C). In addition, FCV-infected cell lysate subjected to H_2_O_2_ treatment for 2 min showed no significant reduction of viral titer in 0.0003–0.3% H_2_O_2_ compared to the untreated control (0%, 7.88 × 10^4^ ± 1.06 × 10^4^ TCID_50_/ml), whereas a significant reduction of viral titer was observed to 1.88 × 10^4^ ± 6.57 × 10^3^ TCID_50_/ml after treatment with 3% H_2_O_2_ (Fig. 6D).

In total, calculation of contribution ratio of the plasma component factors showed 0% heat, UV-A 0%, ONOO^−^ 18.44%, and H_2_O_2_ 0%. This analysis implies that other inactivating factors need to be considered. One possibility is the generation of free radicals during operation of the DBD plasma torch.

**Figure 7:**
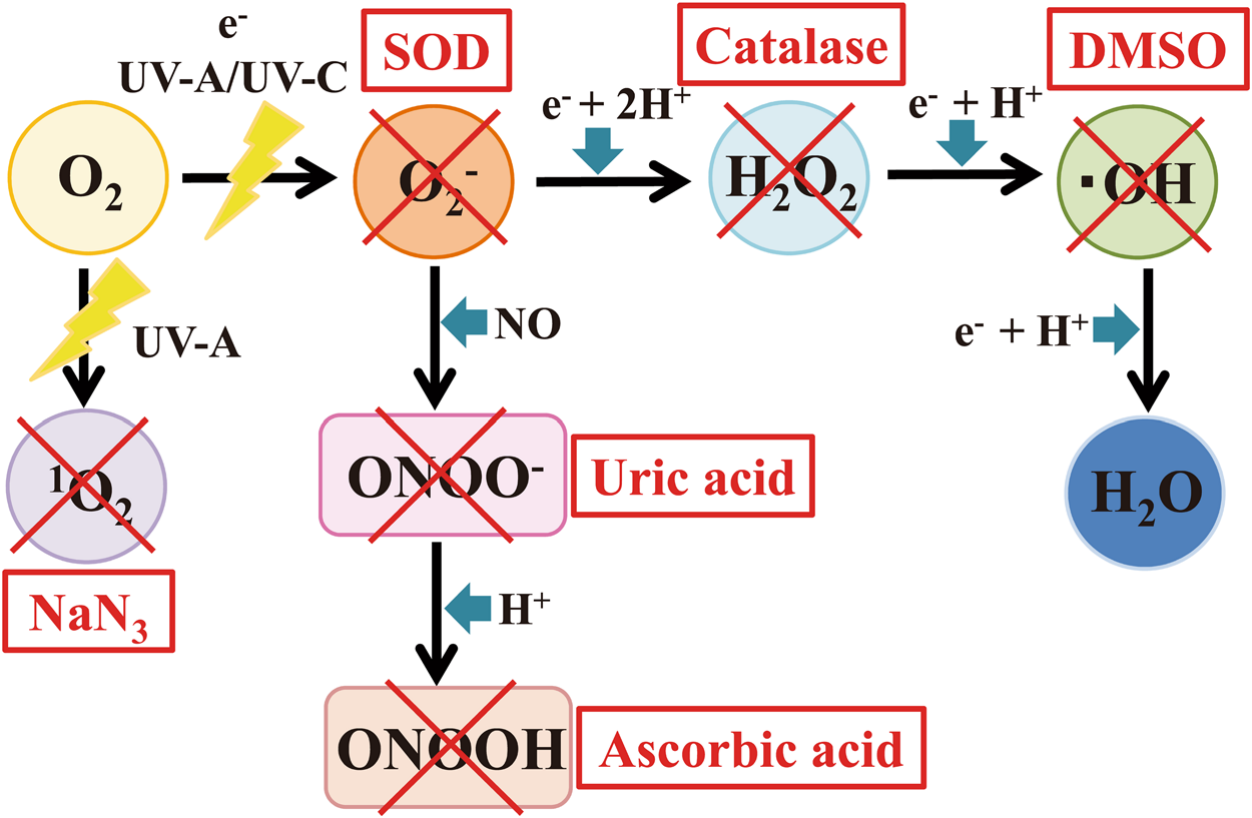
Radical scavengers and reactions. After exposure to UV-A, O_2_ is converted into ^1^O_2_. The ^1^O_2_ is eliminated by sodium azide (NaN_3_), which acts as a scavenger. After exposure to UV-A/UV-C or electrons (e^−^), O_2_ is converted into the superoxide anion radical (·O_2_^−^), which is eliminated by superoxide dismutase (SOD). ·O_2_^−^ is converted into H_2_O_2_ after reaction with e^−^ + 2H^+^. H_2_O_2_ is eliminated by catalase. After the reaction with e^−^ + H^+^, H_2_O_2_ is converted into a hydroxyl radical (·OH). ·OH is eliminated by dimethyl sulfoxide (DMSO). After the reaction with e^−^ + H^+^, ·OH is converted into H_2_O. After the reaction with NO, O_2_^−^ is converted into ONOO^−^, which is eliminated by uric acid. ONOO^−^ is then converted into ONOOH after reaction with H^+^, and ONOOH is eliminated by ascorbic acid.

**Figure 8:**
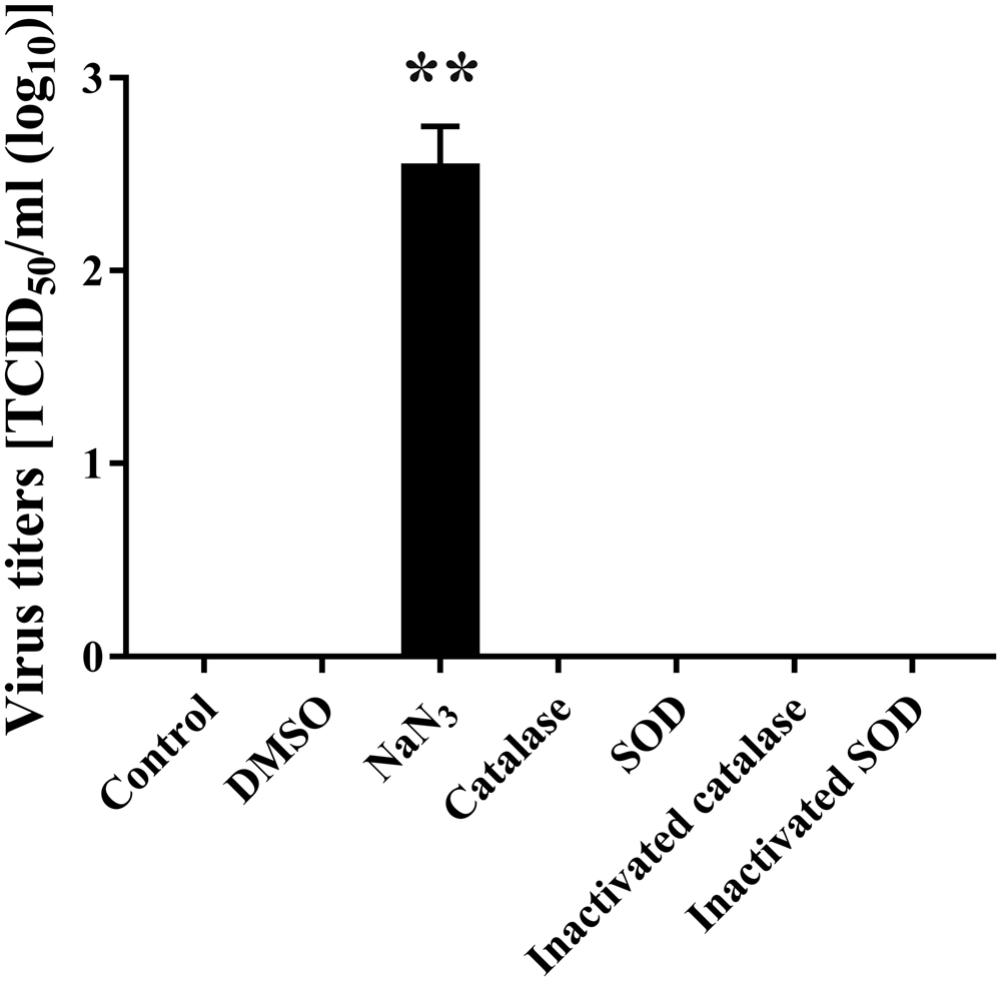
Effect of elimination of ·OH, ^1^O_2_, H_2_O_2_, and ·O_2_^−^ by radical scavengers on viral titer of FCV during operation of the DBD plasma torch. Radical scavengers for ·OH (DMSO), ^1^O_2_ (NaN_3_), H_2_O_2_ (catalase), and ·O_2_^−^ (SOD), as well as inactivated SOD and catalase, were added to the FCV-infected cell lysate. Samples were then exposed to the DBD plasma torch for 2 min, and the viral titer of FCV (TCID_50_) was determined. Zero viral titer means below the detection limit. Differences with **p < 0.01 versus the control were considered significant.

Next, the influence of radical scavengers specific for ·OH, ·O_2_^−^, H_2_O_2_, ^1^O_2_, ONOO^−^, and ONOOH were investigated (Fig. 7) because these radicals are potentially generated during operation of the DBD plasma torch. Because ROS and RNS both have a very short lifetime, plasma treatment was carried out for 2 min under conditions where each radical scavenger was added to FCV-infected cell lysate. The infectivities were then compared in the presence and absence of radical scavengers (Fig. 8). In the presence of radical scavengers for ·OH (DMSO), H_2_O_2_ (Catalase), ·O_2_^−^ (SOD) as well as inactivated SOD and catalase, the infectivity after plasma treatment did not increase. However, in the presence of a radical scavenger for ^1^O_2_ (NaN_3_), the viral titer of the 2 min-plasma treated cell lysate significantly increased as compared with the Control (absence of radical scavenger). Furthermore, the combination of two (Supplemental Fig. 1) or three radical scavengers (Supplemental Fig. 2) also revealed that the presence of radical scavengers for ^1^O_2_ (NaN_3_) significantly increased viral titer. By contrast, however, other radical scavengers did not affect viral titers, suggesting that ^1^O_2_ is the main inactivating factor in addition to ONOO^−^. Furthermore, when the starting viral titer of TCID_50_/ml was 3.58 × 10^4^ ± 1.06 × 10^4^, the addition of sufficient NaN_3_ (10 mM) for complete inhibition of ^1^O_2_ during 1 min of plasma treatment increased the viral titer to 1.05 × 10^3^ ± 2.28 × 10^2^ as compared with 2.65 × 10^2^ ± 1.32 × 10^2^ after 1 min of plasma treatment in the absence of NaN_3_ (data not shown). From these data, the estimated contribution of ^1^O_2_ to FCV inactivation was 2.93%, supporting the idea that ^1^O_2_ is a minor inactivating factor during operation of the DBD plasma torch.

**Figure 9:**
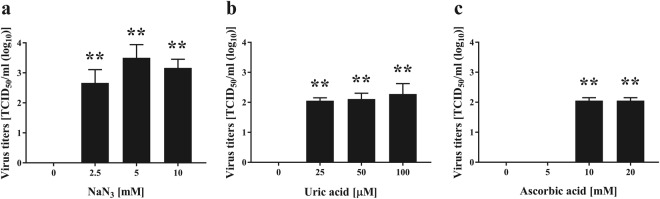
Effect of elimination of ^1^O_2_, ONOO^−^, and ONOOH by radical scavengers on viral titer of FCV during operation of the DBD plasma torch. The indicated concentrations of radical scavengers for ^1^O_2_ (NaN_3_) (**a**), ONOO^−^ (uric acid) (**b**), and ONOOH (ascorbic acid) (**c**) were added to FCV-infected cell lysate. Samples were then exposed to the DBD plasma torch for 2 min, and the viral titer of FCV (TCID_50_) was determined. Zero viral titer means below the detection limit. Differences with **p < 0.01 versus the control (0 mM for NaN_3_ and ascorbic acid; 0 µM for uric acid) were considered significant.

To further confirm the importance of ONOO^−^, increasing concentrations of radical scavengers for ONOO^−^ (uric acid) and ONOOH (ascorbic acid) were added during 2 min of plasma treatment (Fig. 9). Notably, viral titers were significantly higher in the 2-min plasma-treated cell lysates containing radical scavengers than in the control cell lysate exposed to plasma treatment alone.

**Figure 10:**
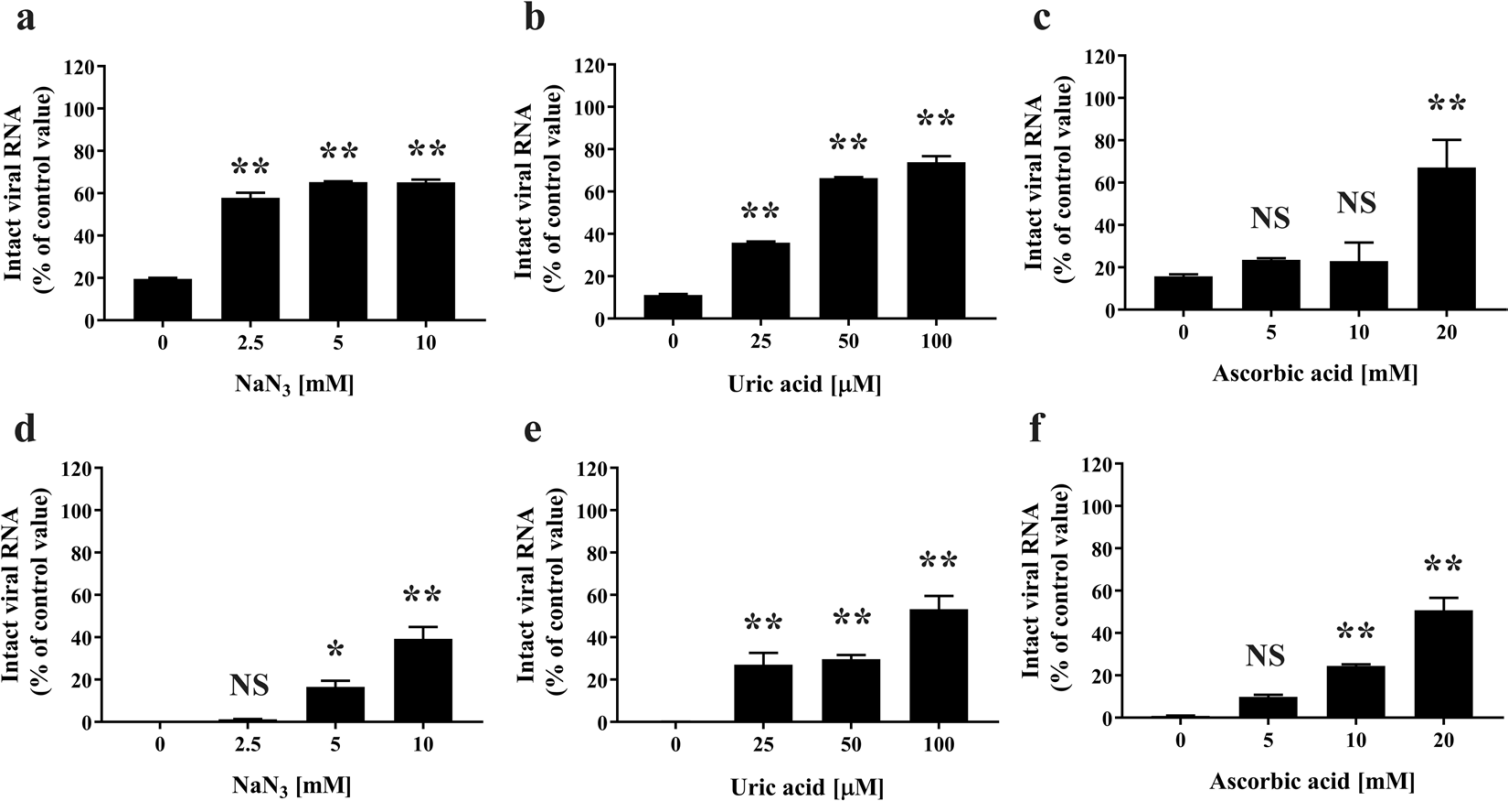
Elimination of ^1^O_2_, ONOO^−^, and ONOOH prevents viral RNA damage of FCV by the DBD plasma torch. FCV-infected cell lysates plus the indicated concentrations of radical scavengers for ^1^O_2_ (NaN_3_) (**a**,**d**), ONOO^−^ (uric acid) (**b**,**e**), and ONOOH (ascorbic acid) (**c**,**f**) were subjected to DBD plasma torch treatment for 2 min. Viral RNA was extracted and the levels of intact viral RNA were analyzed by real-time polymerase chain reaction (PCR) using primers for FCV [(**a**–**c**) FCV-F1 and FCV-R1 or (**d**–**f**) FCV-F2 and FCV-R2] as described in Materials and Methods. Differences with **p < 0.01 or *p < 0.05 versus the control (0 mM for NaN_3_ and ascorbic acid; 0 µM for uric acid) were considered significant; NS means no significance.

Next, to investigate whether ^1^O_2_ and ONOO^−^ are involved in the damage of viral RNA, real-time PCR for FCV viral RNA using primer sets including FCV-F1 and FCV-R1 or FCV-F2 and FCV-R2 was performed after plasma torch treatment in the presence of increasing concentrations of radical scavengers for ^1^O_2_ (NaN_3_), ONOO^−^ (uric acid), and ONOOH (ascorbic acid) (Fig. 10). In this case, the levels of intact viral RNA increased significantly as the concentration of the radical scavengers increased. The amplified DNA was subcloned into T-vector pMD20 and verified by sequencing to correspond to the nonstructural protein of FCV (96–98% identical to Genbank accession number M86379) in the case of primer sets FCV-F1 and FCV-R1 (*N* = 4) or to the VP1 of FCV (95% identical to Genbank accession number AB643784) in the case of primer sets FCV-F2 and FCV-R2 (*N* = 6).

In addition, we performed experiments using rotavirus, which is a non-enveloped RNA virus similar to FCV. Real-time PCR for viral RNA of rotavirus using primer sets for rotavirus VP7 showed a decrease of intact viral RNA after treatment with the DBD plasma torch, suggesting that the DBD plasma torch (Supplemental Fig. 3) also damages the viral RNA of rotavirus. Furthermore, ^1^O_2_, ONOO^−^, and ONOOH are also important for viral RNA damage of rotavirus. Real-time PCR using the above VP7 primer sets showed that intact RNA of rotavirus significantly increased upon addition of radical scavengers, such as NaN_3_, uric acid and ascorbic acid, to the cell lysate of rotavirus-infected cells during operation of the DBD plasma torch (Supplemental Fig. 4).

## Discussion

Recently, there has been significant interest in inactivating human norovirus using non-thermal technologies^6^. Given the practical difficulties of working with human norovirus, many researchers have chosen to use murine norovirus as a surrogate^41,42^. However, recent studies suggest that FCV is more resistant to heat and disinfectant than murine norovirus^36–38^. In addition, FCV has long been used as a surrogate for human norovirus in the evaluation of food preservation and disinfection processes^5,8,32–34,43^. Thus, there is a plethora of information concerning the effect of disinfection treatments on FCV.

Our results suggest that treatment with a DBD plasma torch is an effective means of inactivating FCV. Furthermore, plasma treatment was found to damage the viral RNA of FCV. The FCV infectious titer after plasma treatment for 2 min decreased by at least 99.99%, satisfying the criteria listed for an EPA virucidal agent (at least log_10_ reduction of FCV viral titer)^32^. Therefore, the DBD plasma torch treatment is considered to be an effective virucidal method. In addition, the *D* value of plasma treatment (0.450 min) is markedly shorter than the *D* value for heat treatment of FCV at 56 °C (6.7 min)^36^. This observation further confirms that plasma treatment is an efficient method of inactivation. Indeed, a similar efficient *D* value was previously observed against FCV using a DBD plasma torch employing Ar + 1% O_2_ (0.38 min)^34^.

Previous studies have shown that the major inactivation factors of plasma are UV radiation, H_2_O_2_, and heat, depending on the process gas used^23,25,39,40,44–47^. Aboubakr *et al*. reported that both ^1^O_2_ and ONOOH (peroxynitrous acid) were essential in the inactivation of FCV using a DBD Ar + O_2_ based plasma torch^48^. Moreover, the primary factor in inactivation of herpes simplex virus 1 during exposure to a DBD air-based plasma torch was found to be a reactive chemical species^49^, while those of FCV were ozone (O_3_) and RNS^50^. Taken together, these findings suggest that reactive species, including ROS and RNS, are important in virus inactivation by a plasma torch, and these factors are independent of the source gas.

**Figure 11:**
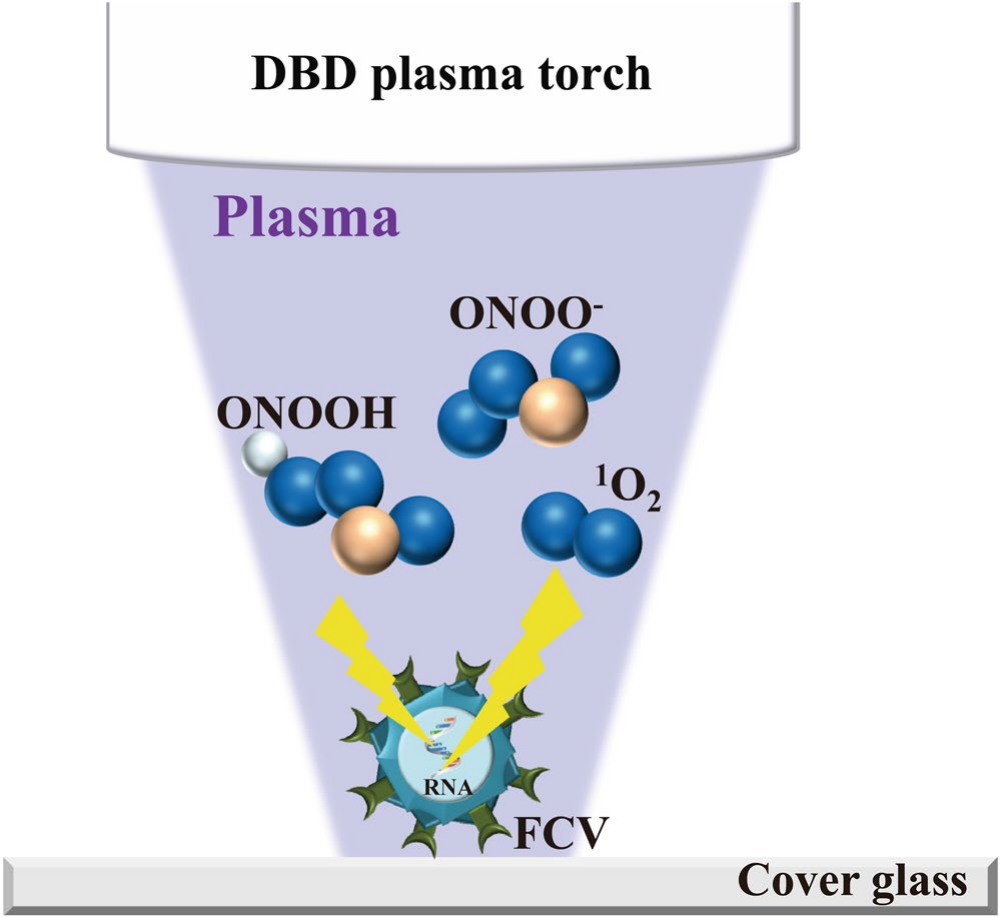
Schematic representation of the mechanism of inactivation of FCV mediated by the DBD plasma torch. During operation of the DBD plasma torch, reactive chemical products such as ^1^O_2_, ONOO^−^, and ONOOH are generated. These reactive chemical products are thought to modify FCV genomic RNA, ultimately leading to inactivation of the virus.

With these background studies in mind, we chose to examine the treatment of FCV with a DBD air-based plasma torch. Because our plasma torch system utilizes air, it is cost-effective compared to plasma torch methods that use other gases such as He, Ar, N_2_ or O_2_. We investigated the effect of individual plasma constituents on FCV as potential inactivating factors in combination with radical scavengers. Our results suggested that heat, UV-A, ·OH, ·O_2_^−^, H_2_O_2_ were not the main inactivating factors, while ONOO^−^ and ^1^O_2_ significantly contributed to FCV inactivation (Fig. 11). Because ONOO^−^ forms ONOOH under acidic conditions, ONOOH may also contribute to FCV inactivation. This idea is supported by our results using ascorbic acid as a radical scavenger for ONOOH. The inactivation contribution ratio of ONOO^−^ was estimated to be 18.44%, indicating that ONOO^−^ had an inactivating effect on FCV during treatment with the DBD plasma torch. In this experiment, we could not directly calculate the contribution ratio of ^1^O_2_ in the plasma because we were unable to measure the concentration of ^1^O_2_. However, our study using radical scavengers indicated that the relative contribution of ^1^O_2_ to FCV inactivation during operation of the DBD plasma torch was 2.93%.

In addition to ^1^O_2_ and ONOO^−^, other free radicals may be generated that contribute to viral inactivation because the sum of the contribution of ^1^O_2_ (2.93%) and ONOO^−^ (18.44%) was only 21.37%. This observation implies the presence of other potential inactivation factors. The possibility of other inactivation factors is consistent with the results of a previous report^48^, which highlighted the essential role of ONOOH in addition to ^1^O_2_ for the inactivation of FCV using a DBD Ar + O_2_ based plasma torch. The same study also showed that ^1^O_2_ reacts with histidine residues in the FCV capsid protein to oxidize them^48^, suggesting a possible mechanism by which ^1^O_2_ contributes to FCV inactivation. The authors proposed that oxidization of tryptophan and methionine residues in the viral proteins may also be involved in the virucidal activity. However, the effect of DBD plasma on FCV genomic RNA was not examined.

The present study has confirmed that plasma treatment causes damage to FCV genomic RNA. Moreover, these findings infer that damage to the viral genome is involved in the reduction of infectivity. Indeed, our findings are consistent both with the observation that the DBD plasma torch destroys the genomic DNA of *Helicobacter pylori*^39^, and with previous studies showing that a nitrogen plasma destroys the genomic RNA of influenza virus^25^ and genomic DNA of adenovirus^26^. Furthermore, other plasma sources have been reported to damage the DNA of bacteriophage^51^ and adenovirus^52^. However, as FCV did not show any reduction in viral titer after UV-A exposure at the levels of UV-A generated from DBD plasma torch within 2 min, FCV may be relatively resistant to UV-A compared to other viruses such as influenza virus^44^. The present study suggests that viral RNA damage caused by ^1^O_2_ and ONOO^−^, as well as ONOOH, are the predominant mechanisms by which FCV is inactivated following treatment with the DBD plasma torch of FCV-infected cell lysate (Fig. 11) as well as rotavirus-infected cell lysate (Supplemental Figs 3 and 4). In Fig. 9, a significant increase of viral titer of FCV was observed in the presence of 10 mM ascorbic acid compared to the absence of radical scavenger (Control). However, no significant difference was observed in intact viral RNA of FCV compared to the control under the same conditions. Therefore, there is a discrepancy between virus titer and intact viral RNA of FCV in the presence of 10 mM ascorbic acid. This discrepancy may suggest the presence of other inactivating processes besides RNA damage of FCV. One possibility is viral protein damage such as oxidation. Thus, the precise damage to the viral RNAs and proteins of FCV induced by plasma torch treatment remains unclear. Further studies are required to understand the mechanism of FCV inactivation resulting from DBD plasma torch treatment.

Finally, it should be noted that although FCV is the representative surrogate of human norovirus and well-known to be resistant to disinfecting agents, the results cannot be extrapolated to human norovirus without verification. Thus, future studies on plasma inactivation using human norovirus are required. In addition, further optimization and scale-up of the plasma instrument may be necessary for the practical use of this technology.

## Methods

### Virus and cells

FCV F9 strain [VR-782; ATCC (American type Culture Collection, Manassas, VA, USA)] was used as the test virus. Crandell-Rees feline kidney-cell (CRFK) cells (CCL-94; ATCC) were used for the infection assays.

### Preparation of cell lysate

CRFK cells were maintained in minimum essential medium (MEM) supplemented with 10% fetal bovine serum (FCS) (JRH Biosciences Inc., Saint Louis, MO, USA). Penicillin-streptomycin (PS) was added to MEM by 100 units/ml of penicillin, 100 μg/ml of streptomycin (Nakalai Tesque, Kyoto, Japan). CRFK cells (3 × 10^5^ cells) were grown in 100 mm dishes and adsorbed with FCV at a MOI (multiplicity of infection) of 0.1 for 1 h. The medium was then exchanged with 2% FCS and PS-supplemented MEM medium and cultured at 37 °C under 5% CO_2_ for 3 days. The cultured FCV-infected cells were suspended in phosphate buffered saline (PBS) (Life Technologies, Carlsbad, CA, USA), centrifugally washed three times, and freeze-thawed at −80 °C. Thereafter, the collected cells were disrupted by passage through a 28G injection needle and suspended in PBS to prepare a FCV-infected cell lysate.

### DBD plasma torch

A torch used for plasma generation was previously fabricated using a ceramic tube (Al_2_O_3_) with a length of 100 mm and an inner and outer diameter of 4 mm and 6 mm, respectively^39^. Copper tape (0.08 mm thickness and 60 mm length) was wound around the tube as an earth electrode (Fig. 1). Stainless steel mesh (SUS304) was placed inside the tube to act as a high voltage electrode. The two electrodes were connected to a high-voltage power supply unit with low frequency (10 kV peak-to-peak, 10 kHz). The flow rate of air was maintained at 3.5 L/min using an air pump (Suishin SSPP-2S, Suisaku Co., Tokyo, Japan). Aliquots (20 μl) of cell lysate on a cover glass (Matsunami Glass Ind., Ltd., Osaka, Japan) were subjected to DBD plasma treatment. In all cases, the distance from the torch tip to the liquid surface on the cover glass was 20 mm.

### Viral titration assay

Samples were 10-fold-diluted with PBS and added to CRFK cells (7.5 × 10^3^ cells/well) seeded on a 96-well microtiter plate. Cells were then cultivated at 37 °C under 5% CO_2_ conditions for 3 days. A cytopathic effect (CPE) was observed and TCID_50_ (median tissue culture infectious dose) was calculated from the CPE based on the Behrens-Kärber method^53^.

### Heat treatment of FCV

Aliquots (20 μL) of FCV-infected cell lysate were placed onto a cover glass and incubated at various temperatures (35–70 °C) for 2 min using a heat block BI-516S (Astec Co., Ltd., Fukuoka, Japan). The treated and untreated samples were then analyzed using the viral titration assay as described above.

### Viral RNA extraction and real-time polymerase chain reaction (PCR)

Viral RNA was extracted using a QIAamp Viral RNA mini kit (Qiagen, Hilden, Germany) according to the manufacturer’s instructions. The RNA was then eluted into 60 μl of nuclease-free water. The RNA was transcribed with PrimeScriptII 1st strand cDNA Synthesis kit (Takara Bio Inc., Otsu, Japan) and random primers to make cDNA using the following temperature regime: 65 °C for 5 min, 4 °C for 5 min, 42 °C for 60 min, and 95 °C for 5 min. The resultant cDNA was analyzed by real-time PCR using SYBR Premix Ex Taq II (Tli RNase H plus) (Takara Bio Inc., Shiga, Japan) according to the manufacturer’s instructions (Stratagene, La Jolla, CA). Briefly, the real-time PCR components included SYBR Premix Ex Taq II, and the forward and reverse target gene primers designed to the nonstructural protein of FCV i.e. Genbank accession number M86379:

Forward primer (FCV-F1): 5′-TAATTCGGTGTTTGATTTGGCCTGGGCT-3′;Reverse primer (FCV-R1): 5′-CATATGCGGCTCTGATGGCTTGAAACTG-3′,

and the forward and reverse target gene primers designed to VP1 of FCV i.e. Genbank accession number AB643784:

Forward primer (FCV-F2): 5′-GTTGGTGGGGTTATCGCTGA-3′;

Reverse primer (FCV-R2): 5′-CCCACTCAGAGTCAACGCTT-3′.

Real-time PCR was performed using a Thermal Cycler Dice Real Time System (Takara Bio Inc.). The cycling program included initial denaturation at 95 °C for 30 sec followed by 40 cycles of 95 °C for 30 sec and 60 °C for 30 sec. Each reaction was carried out in quadruplicate and the results were analyzed using Thermal Cycler Dice Realtime System Single software (Takara Bio Inc.). The relative DNA levels of each sample were compared with serially diluted viral cDNA and estimated using the standard curve of diluted viral cDNA versus value of Ct (threshold cycle). PCR specificity was verified by dissociation curve analysis of the amplified DNA fragments of step 1 (95 °C/15 sec), step 2 (60 °C/30 sec), and step 3 (95 °C/15 sec). The amplified products were subjected to DNA sequencing after subcloning into Takara T-Vector pMD20 (Takara Bio Inc.), with an ABI373OXL Genetic Analyzer (Applied Biosystems, Foster City, CA, USA) in order to verify the amplified products.

### Indirect immunofluorescence Assay

Detection of FCV in FCV-infected CRFK cells was performed by indirect immunofluorescence assays. After incubation with samples for 1 day, cells were fixed with 4% paraformaldehyde for 20 min and cold methanol for 10 min. Cells were blocked with 3% bovine serum albumin (BSA) for 30 min and incubated with anti-FCV antibody (ab33990 [FCV1-43]; Abcam, Cambridge, UK) at 1: 100 dilution in PBS for 1 h at 37 °C. Cells were subsequently labeled with fluorescein isothiocyanate (FITC)-labeled donkey anti-mouse IgG (Jackson ImmunoResearch Laboratories, Inc., West Grove, PA, USA) at 1: 200 dilution by PBS for 30 min at 37 °C. The stained cell monolayer was visualized under a fluorescence microscope (Biozero BZ-8100; Keyence, Osaka, Japan).

### Measurement of optical emission spectra

A multichannel spectrophotometer (MCPD-7000; Otsuka Electronics Co. Ltd. Osaka, Japan) attached to a fiber probe was used for measuring the emission of 300–1100 nm during DBD plasma torch operation. Spectra were collected for 400 msec with 20 times integration time.

### Irradiation of FCV with UV-A

Aliquots (20 μL) of FCV-infected cell lysate were dropped onto a cover glass (Matsunami Glass Ind., Ltd.) and then subjected to UV-A irradiation as follows. The spots were irradiated with UV-A using a UV transilluminator UVGL-58 (UVP; Upland, CA, USA) for 0–30 min. The distance between the drops and UV transilluminator was maintained at 20 mm. The energy (mJ/cm^2^) of UV-A was estimated on the basis of color changes to UV indicators (UV label-S) (NiGK Corporation, Tokyo, Japan). The treated FCV-infected cell lysate samples were used for viral titration assays as described above.

### Hydrogen peroxide (H_2_O_2_) treatment of FCV

The effect of H_2_O_2_ (Wako Pure Chemical Industries Ltd., Osaka, Japan) on FCV was determined as follows. Aliquots (10 μL) of FCV-infected cell lysate were incubated with an equal volume of various concentrations (0–3%) of H_2_O_2_ at room temperature for 2 min. The resultant samples were then subjected to the viral titration assay as described above.

### Measurement of UV using a UV chemical indicator

UV energy generated during exposure to the plasma torch was determined using a test strip (UV label-S; NiGK Corporation). The change in colour of the UV label-S test strip was measured after scanning to generate an image, which was converted to an RGB (Red, Green, and Blue) code. The precise value was calculated on the basis of a standard curve developed from the RGB code of a reference.

### Measurement of H_2_O_2_ using a chemical indicator strip

A chemical indicator strip (Quantofix peroxide 100 test strip; Macherey-Nagel GmbH & Co. KG, Duren, Germany) was used to determine the concentration of H_2_O_2_ generated during exposure to the plasma torch. Specifically, 20 µL of PBS was dropped onto the indicator strip and subjected to the plasma torch treatment. The indicator strips were positioned at a distance of 20 mm from the DBD plasma torch top. After the plasma torch treatment, changes in the colour of the test strip were measured after scanning to generate an image, which was subsequently converted to an RGB code. The precise value was calculated on the basis of a standard curve developed from the RGB code of a reference strip.

### Thermography

The temperature of a spot of cell lysate on a cover glass during plasma treatment was determined using infrared thermography (FLIR i5; FLIR systems, Wilsonville, OR, USA) as described previously^24^.

### ONOO^−^ measurements

NO_2_^−^ and NO_3_^−^ was measured using the NO_2_^−^/NO_3_^−^ assay kit-CII Colorometric Griess Reagent kit (Dojindo, Kumamoto, Japan) in accordance with the company’s instructions. The kit is based on the method of Griess^54^ where peroxynitrite (ONOO^−^) is finally converted into NO_2_^−^. The concentration of NO_2_^−^ in samples was calculated from the standard curve of NO_2_^−^ versus absorbance at 540 nm. ONOO^−^ concentration was estimated from the standard curve of ONOO^−^ versus NO_2_^−^.

### ONOO^−^ treatment of FCV

Aliquots (10 μL) of ONOO^−^ solution (Dojindo) adjusted to 0.25–8.0 mM with PBS were mixed with 10 μL of FCV-infected cell lysate and incubated at room temperature for 2 min. Samples were quickly diluted with 400 μL of PBS and TCID_50_ was measured as described above.

### Elimination of radicals by radical scavengers

The plasma treatment was performed in the presence of the following radical scavengers for eliminating ROS and RNS. Dimethyl sulfoxide (DMSO) at 10 mM (Cat. No. 13407-45, Nakalai Tesque) was used for elimination of hydroxyl radicals (·OH), while superoxide dismutase (SOD) (Cat. No. 198-11283, Wako Pure Chemical Industries Ltd.) at 300 U/ml was used for the elimination of superoxide anion radicals (·O_2_^−^). Catalase (Cat. No. C1345-1G, Sigma-Aldrich, St Louis, MO, USA) at 300 U/ml was used for elimination of H_2_O_2_, while 10 mM of sodium azide (NaN_3_) (Cat. No. 195-11092, Wako Pure Chemical Industries Ltd.) was used for the elimination of singlet oxygen species (^1^O_2_). Uric acid (Cat. No. 217-01612, Wako Pure Chemical Industries Ltd.) was used at 0–100 μM for the elimination of ONOO^−^. *L*(+)-ascorbic acid (Cat. No. 014-04801, Wako Pure Chemical Industries Ltd.) was used at 0–20 mM for the elimination of peroxynitrous acid (ONOOH). After treatment, 20-μL aliquots were mixed with 200 μL of PBS and subjected to the viral titration assay and real-time PCR.

### Calculation of *D* value (Decimal reduction time)

The treatment time *(D* value) to lower the virus infectivity value to 1/10 was obtained using the following formula, which is modified from a previous report^12^.

$$D\,{\rm{value}}={{\rm{\Delta }}\mathrm{log}}_{10}\,{\rm{V}}/{\rm{\Delta }}t$$

where Δlog_10_V: logarithm of the change in the viral titer, Δt: change in time, V: viral titer.

### Determining the contribution ratio of individual disinfecting factors generated during operation of the plasma torch

Contribution ratio was defined as the proportion of decreased viral titer induced by an individual disinfecting factor to the overall decreased viral titer induced by treatment with the plasma torch.

The contribution ratio was calculated using the following equation:

$${\rm{Contribution}}\,{\rm{ratio}}( \% )=(r/R)\times 100,$$

where *r* represents the logarithm of the reduced viral titer of FCV treated with an individual disinfecting factor (e.g. UV-A, ONOO^−^, and H_2_O_2_) generated during operation of the plasma torch, while *R* represents the logarithm of the reduced viral titer of FCV-treated with the plasma torch.

These terms are defined as follows:

$$\begin{array}{rcl}r & = & -\mathrm{log}\,{{\rm{E}}}_{{\rm{t}}}/{{\rm{E}}}_{0}\\ R & = & -\mathrm{log}\,{{\rm{P}}}_{{\rm{t}}}/{{\rm{P}}}_{0}\end{array}$$

where E_0_ is the viral titer of untreated FCV (0 min, individual disinfecting factor), while P_0_ is the viral titer of untreated FCV (0 min, no treatment with the plasma torch). E_t_ is the viral titer of FCV after treatment with an individual disinfecting factor generated during operation of the plasma torch, while P_t_ is the viral titer of FCV after treatment with the plasma torch.

### Statistical analysis

The results are the mean ± standard error of mean (SEM) of experiments conducted in at least triplicate. The statistical analysis of significant difference was performed by non-repeated analysis of variance (ANOVA) followed by Bonferroni’s multiple comparison test. The analysis was carried out using the GraphPad Prism 5 software (GraphPad Prism Software Inc., La Jolla, CA, USA).

## Electronic supplementary material

Supplementary Information

## References

[1] Patel MM, Hall AJ, Vinje J, Parashar UD (2009). Noroviruses: a comprehensive review. J. Clin. Virol..

[2] CDC. US trends and outbreaks, http://www.cdc.gov/norovirus/trends-outbreaks.html/ (2016).

[3] Melhem NM (2016). Norovirus vaccines: Correlates of protection, challenges and limitations. Hum. Vaccin. Immunother..

[4] Teunis PF (2008). Norwalk virus: how infectious is it?. J. Med. Virol..

[5] Doultree JC, Druce JD, Birch CJ, Bowden DS, Marshall JA (1999). Inactivation of feline calicivirus, a Norwalk virus surrogate. J. Hosp. Infect..

[6] Cook N, Knight A, Richards GP (2016). Persistence and Elimination of Human Norovirus in Food and on Food Contact Surfaces: A Critical Review. J. Food Prot..

[7] US-EPA. List G: EPA registered hospital disinfectants effective against norovirus (Norwalk-like virus), https://www.epa.gov/sites/production/files/2016-06/documents/list_g_norovirus.pdf/ (2016).

[8] Sanekata T (2010). Evaluation of the antiviral activity of chlorine dioxide and sodium hypochlorite against feline calicivirus, human influenza virus, measles virus, canine distemper virus, human herpesvirus, human adenovirus, canine adenovirus and canine parvovirus. Biocontrol Sci.

[9] Tung G, Macinga D, Arbogast J, Jaykus LA (2013). Efficacy of commonly used disinfectants for inactivation of human noroviruses and their surrogates. J. Food Prot..

[10] Johns CK (1934). Germicidal power of sodium hypochlorite - Effect of addition of alkali. Ind. Eng. Chem..

[11] Whitehead K, McCue KA (2010). Virucidal efficacy of disinfectant actives against feline calicivirus, a surrogate for norovirus, in a short contact time. Am. J. Infect. Control.

[12] Sakudo, A. & Shintani, H. *Sterilization and Disinfection by Plasma: Sterilization Mechanisms, Biological and Medical Applications (Medical Devices and Equipment)* (Nova Science Publishers, 2016).

[13] CDC. Preventing norovirus infection, https://www.cdc.gov/norovirus/preventing-infection.html/ (2016).

[14] Horvitz S, Cantalejo MJ (2014). Application of ozone for the postharvest treatment of fruits and vegetables. Crit. Rev. Food Sci. Nutr..

[15] Rice, R. G., Graham, D. M. & Sopher, C. D. Case studies of ozone in agri-food applications in *Nonthermal Processing Technologies for Food* (ed. Zhang, H. Q., Barbosa-Cánovas, G. V., Balasubramaniam, V.M., Dunne, C. P., Farkas, D. F. & Yuan, J. T. C.) 314–341 (Wiley-Blackwell, 2010).

[16] Cutler TD, Zimmerman JJ (2011). Ultraviolet irradiation and the mechanisms underlying its inactivation of infectious agents. Anim. Health Res. Rev..

[17] Sommers, C. & Fan, X. Irradiation of ground beef and fresh produce in *Nonthermal processing technologies for food*. (ed. Zhang, H. Q. *et al*.) 236–248 (Wiley-Blackwell, 2010).

[18] Kramer B, Wunderlich J, Muranyi P (2017). Recent findings in pulsed light disinfection. J. Appl. Microbiol..

[19] Considine KM, Kelly AL, Fitzgerald GF, Hill C, Sleator RD (2008). High-pressure processing–effects on microbial food safety and food quality. FEMS Microbiol. Lett..

[20] Khan SI (2016). Eradication of multidrug-resistant pseudomonas biofilm with pulsed electric fields. Biotechnol. Bioeng..

[21] Grigelmo-Miguel, N., Soliva-fortuny, R., Barbosa-Cánovas, G. V. & Martín-Belloso, O. Use of oscillating magnetic fields in food preservation in *Nonthermal processing technologies for food* (ed. Zhang, H. Q. *et al*.) 222–235 (Wiley-Blackwell, 2010).

[22] Feng, H. & Yang, W. Ultrasonic processing, In N*onthermal processing technologies for food* (ed. Zhang, H. Q. *et al*.) 135–154 (Wiley-Blackwell, 2010).

[23] Maeda K, Toyokawa Y, Shimizu N, Imanishi Y, Sakudo A (2015). Inactivation of S*almonella* by nitrogen gas plasma generated by a static induction thyristor as a pulsed power supply. Food Control.

[24] Toyokawa Y, Yagyu Y, Misawa T, Sakudo A (2017). A new roller conveyer system of non-thermal gas plasma as a potential control measure of plant pathogenic bacteria in primary food production. Food Control.

[25] Sakudo A, Shimizu N, Imanishi Y, Ikuta K (2013). N_2_ gas plasma inactivates influenza virus by inducing changes in viral surface morphology, protein, and genomicRNA. Biomed. Res. Int..

[26] Sakudo A, Toyokawa Y, Imanishi Y (2016). Nitrogen gas plasma generated by a static induction thyristor as a pulsed power supply inactivates adenovirus. PLoS One.

[27] Malik YS, Maherchandani S, Allwood PB, Goyal SM (2005). Evaluation of animal origin cell cultures for i*n vitro* cultivation of noroviruses. J. Appl. Res. Clin. Exp. Ther..

[28] Straub TM (2007). *In vitro* cell culture infectivity assay for human noroviruses. Emerg. Infect. Dis..

[29] Jones MK (2014). Enteric bacteria promote human and mouse norovirus infection of B cells. Science.

[30] Ettayebi K (2016). Replication of human noroviruses in stem cell-derived human enteroids. Science.

[31] Costantini V (2018). Human norovirus replication in human intestinal enteroids as model to evaluate virus inactivation. Emerg. Infect. Dis..

[32] US-EPA. Initial virucidal effectiveness test using feline calicivirus as surrogate for norovirus, https://www.epa.gov/sites/production/files/2015-09/documents/fcv1_initial_surf_pcol.pdf/ (2015).

[33] Butot S, Putallaz T, Sanchez G (2008). Effects of sanitation, freezing and frozen storage on enteric viruses in berries and herbs. Int. J. Food Microbiol..

[34] Aboubakr HA (2015). Virucidal effect of cold atmospheric gaseous plasma on feline calicivirus, a surrogate for human norovirus. Appl. Environ. Microbiol..

[35] D’Souza DH (2006). Persistence of caliciviruses on environmental surfaces and their transfer to food. Int. J. Food Microbiol..

[36] Cannon JL (2006). Surrogates for the study of norovirus stability and inactivation in the environment: A comparison of murine norovirus and feline calicivirus. J. Food Prot..

[37] Sattar SA, Ali M, Tetro JA (2011). *In vivo* comparison of two human norovirus surrogates for testing ethanol-based handrubs: the mouse chasing the cat!. PLoS One.

[38] Park GW (2010). Comparative efficacy of seven hand sanitizers against murine norovirus, feline calicivirus, and GII.4 norovirus. J. Food Prot..

[39] Sakudo A, Miyagi H, Horikawa T, Yamashiro R, Misawa T (2018). Treatment of *Helicobacter pylori* with dielectric barrier discharge plasma causes UV induced damage to genomic DNA leading to cell death. Chemosphere.

[40] Moisan M (2001). Low-temperature sterilization using gas plasmas: a review of the experiments and an analysis of the inactivation mechanisms. Int. J. Pharm..

[41] Hirneisen KA, Kniel KE (2013). Comparing human norovirus surrogates: murine norovirus and Tulane virus. J. Food Prot..

[42] Wobus CE (2004). Replication of Norovirus in cell culture reveals a tropism for dendritic cells and macrophages. PLoS Biol..

[43] Tree JA, Adams MR, Lees DN (2005). Disinfection of feline calicivirus (a surrogate for Norovirus) in wastewaters. J. Appl. Microbiol..

[44] Sakudo A, Misawa T, Shimizu N, Imanishi Y (2014). N_2_ gas plasma inactivates influenza virus mediated by oxidative stress. Front Biosci (Elite Ed).

[45] Laroussi M (2005). Low temperature plasma-based sterilization: overview and state-of-the-art. Plasma Processes Polym..

[46] Laroussi M, Leipold F (2004). Evaluation of the roles of reactive species, heat, and UV radiation in the inactivation of bacterial cells by air plasmas at atmospheric pressure. Int. J. Mass Spectrom..

[47] Bol’shakov AA (2004). Radio-frequency oxygen plasma as sterilization source. AIAA J.

[48] Aboubakr HA, Gangal U, Youssef MM, Goyal SM, Bruggeman PJ (2016). Inactivation of virus in solution by cold atmospheric pressure plasma: identification of chemical inactivation pathways. J. Phys. D Appl. Phys..

[49] Alekseev O, Donovan K, Limonnik V, Azizkhan-Clifford J (2014). Nonthermal dielectric barrier discharge (DBD) plasma suppresses herpes simplex virus type 1 (HSV-1) replication in corneal epithelium. Trans. Vis. Sci. Technol..

[50] Nayak G, Aboubakr HA, Goyal SM, Bruggeman PJ (2018). Reactive species responsible for the inactivation of feline calicivirus by a two-dimensional array of integrated coaxial microhollow dielectric barrier discharges in air. Plasma Process Polym..

[51] Yasuda H, Miura T, Kurita H, Takashima K, Mizuno A (2010). Biological evaluation of DNA damage in bacteriophages inactivated by atmospheric pressure cold plasma. Plasma Process Polym..

[52] Zimmermann JL (2011). Effects of cold atmospheric plasmas on adenoviruses in solution. J. Phys. D Appl. Phys..

[53] Kärber G (1931). 50% end point calculation. Arch. Exp. Pathol. Pharmacol..

[54] Griess P (1879). Bemerkungen zu der abhandlung der H.H. Weselsky und Benedikt“Ueber einige azoverbindungen.”. Chem. Ber..

