# Supplementary material for: Key role of singlet oxygen and peroxynitrite in viral RNA damage during virucidal effect of plasma torch on feline calicivirus
Source: Sci Rep. 2018 Dec 18;8:17947. doi: 10.1038/s41598-018-36779-1 (PMC6298994; doi:10.1038/s41598-018-36779-1)
Supplement: Supplementary file 1 — Supplementary Information [file 41598_2018_36779_MOESM1_ESM.pdf]

Supplementary Information

Key role of singlet oxygen and peroxynitrite in viral RNA damage during virucidal effect of plasma torch on feline calicivirus

Risa Yamashiro[1], Tatsuya Misawa[2], and Akikazu Sakudo[1]*

[1]Laboratory of Biometabolic Chemistry, School of Health Sciences, University of the Ryukyus, Nishihara, Okinawa 903-0215, Japan

[2]Department of Electrical and Electronic Engineering, Faculty of Science and Engineering, Saga University, Saga 840-8502, Japan

*Correspondence and requests for materials should be adressed to A.S. (e-mail: akikazusakudo@gmail.com)

Postal address: Laboratory of Biometabolic Chemistry, School of Health Sciences, Faculty of Medicine, University of the Ryukyus, 207 Uehara, Nishihara, Okinawa 903-0215, Japan. Tel/Fax:+81-98-895-1252

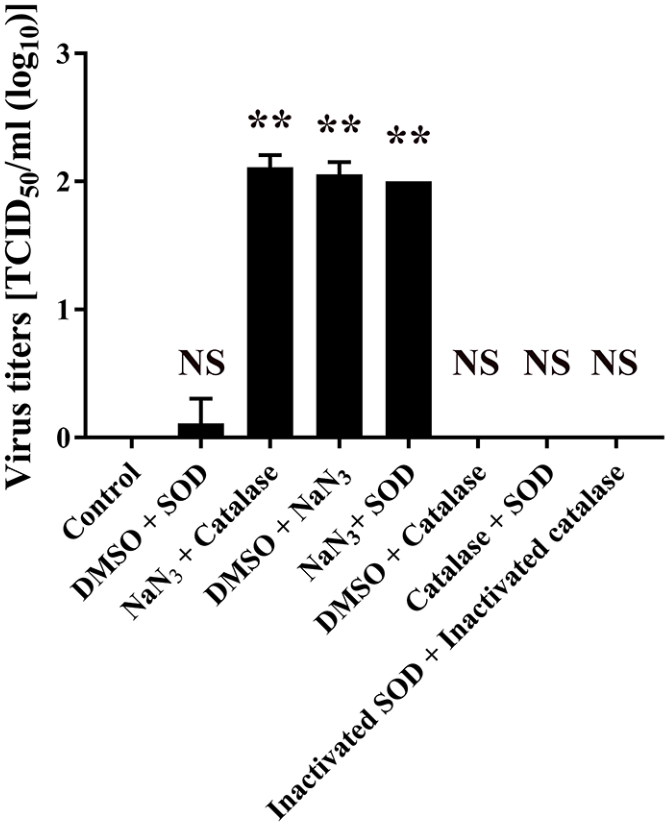

Supplemental Fig. 1 Effect of elimination of free radicals by a mixture of two radical scavengers on viral titer of FCV during operation of the DBD plasma torch

A combination of two radical scavengers (DMSO, SOD, NaN_3, or catalase) as well as inactivated SOD and calatase were added to the FCV-infected cell lysate. Samples were exposed to the DBD plasma torch for 2 min and the viral titer of FCV ($TCID_{50}$) determined. Zero in virus titer means below the detectable limit. Differences where $p<0.01$(**) versus Control were considered significant, while NS means no significance.

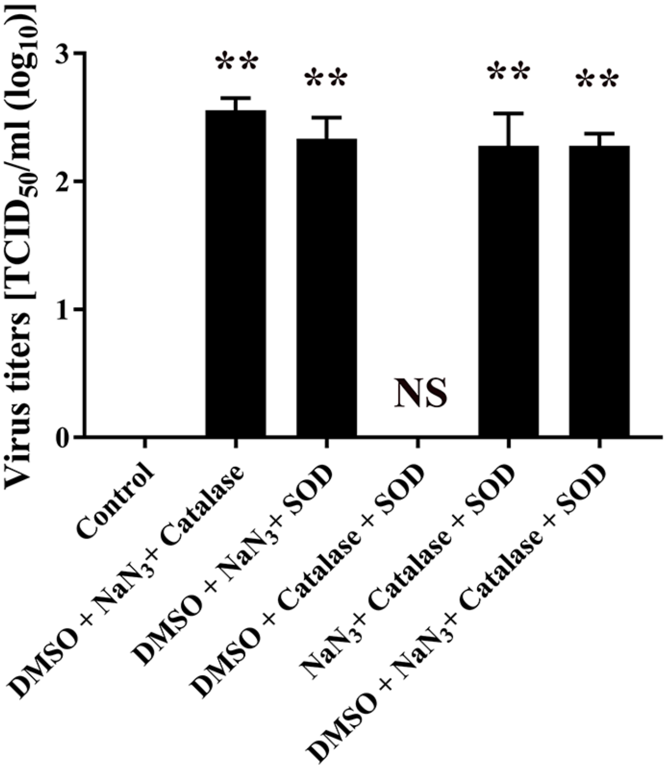

Supplemental Fig. 2 Effect of elimination of free radicals by a mixture of three radical scavengers on viral titer of FCV during operation of the DBD plasma torch

A combination of three radical scavengers (DMSO, SOD, NaN_3, or catalase) as well as inactivated SOD and calatase were added to the FCV-infected cell lysate. Samples were exposed to the DBD plasma torch for 2 min before determining the viral titer of FCV ($TCID_{50}$). Zero in virus titer means below the detectable limit. Differences where $p<0.01$(**) versus Control were considered significant, while NS means no significance.

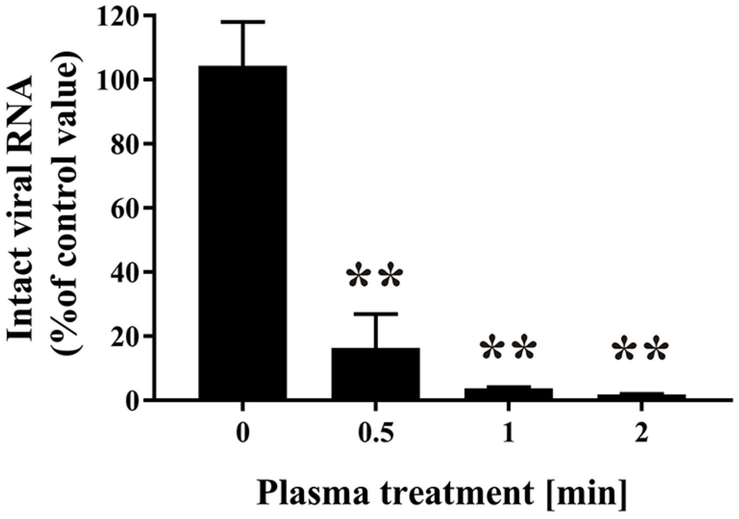

Supplemental Fig. 3 Quantitative analysis of rotavirus RNA after DBD plasma torch treatment

Rotavirus Wa strain [VR-2018; ATCC (American type Culture Collection, Manassas, VA, USA)]-infected cell lysates of MA104 cells were subjected to DBD plasma torch treatment for the indicated time (min). Viral RNA was extracted and subjected to a reverse transcription (RT) reaction. The levels of intact viral RNA were then analyzed by real-time polymerase chain reaction (PCR) using primers specific for rotavirus (i.e. forward and reverse primers: 5'- GTA CCG TGA AAG TGT GTC CG-3'; 5'- TCC CAT CAA CGA CAT CCA CT-3', respectively). Differences where $p<0.01$(**) versus control (0 min) were considered significant. The real-time PCR product (126 bp) was confirmed to be viral coat protein VP7 of human rotavirus A Wa strain (identical to Genbank accession number FJ423153 100%) by DNA sequencing after subcloning into Takara T-Vector pMD20 (Takara Bio Inc.), with an ABI373OXL Genetic Analyzer (Applied Biosystems).

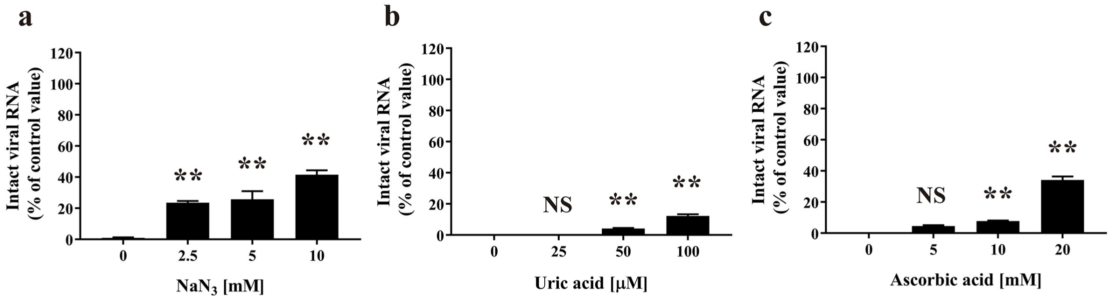

Supplemental Fig. 4 Elimination of $^1O_2$, $ONOO^-$, and ONOOH prevents viral RNA damage of rotavirus by the DBD plasma torch

Rotavirus Wa strain-infected cell lysates of MA104 cells plus the indicated concentrations of radical scavengers for $^1O_2$ (NaN_3) (a), $ONOO^-$ (uric acid) (b), and ONOOH (ascorbic acid) (c) were subjected to DBD plasma torch treatment for 2 min. Viral RNA was extracted and subjected to reverse transcription. The levels of intact viral RNA were then analyzed by real-time PCR using specific primers for rotavirus as described in Supplemental Fig. 3. Differences with $p<0.01$(**) versus the control (no NaN_3, ascorbic acid or uric acid) were considered significant; NS means no significance.
